# Expression Pattern of G-Protein-Coupled Estrogen Receptor in Myometrium of Uteri with and without Adenomyosis

**DOI:** 10.1155/2017/5974693

**Published:** 2017-10-04

**Authors:** Jin-Jiao Li, Hua Duan, Sha Wang, Fu-Qing Sun, Lu Gan, Yi-Qun Tang, Qian Xu, Tin-Chiu Li

**Affiliations:** ^1^Department of Gynecology Minimally Invasive Center, Beijing Obstetrics and Gynecology Hospital, Capital Medical University, Beijing 100006, China; ^2^Department of Obstetrics and Gynecology, The Chinese University of Hong Kong, Shatin, Hong Kong

## Abstract

**Objective:**

To compare the expression of G-protein-coupled estrogen receptor (GPER) in the junctional zone and outer myometrium of the proliferative and secretory phases of women with and without adenomyosis.

**Methods:**

A total of 76 women were included in this study, 42 with adenomyosis (proliferative phase, *n* = 23; secretory phases, *n* = 19) and 34 controls (proliferative phase, *n* = 16; secretory phases, *n* = 18). Protein and total RNA were extracted from the junctional zone (JZ) and outer myometrium (OM). GPER protein and mRNA expression levels were evaluated by the use of western blotting and real-time quantitative polymerase chain reaction (RT-qPCR).

**Results:**

The expression of GPER protein and mRNA in women with adenomyosis was significantly higher than that of control subjects, both in the junctional zone and in the outer myometrium and both in the proliferative and in the secretory phases.

**Conclusion:**

The significant and consistent increase in GPER expression in adenomyosis compared with control subjects, regardless of whether it was in the proliferative or secretory phases and regardless of whether it was in the JZ or OM, suggests that GPER plays an important role in the pathogenesis of the adenomyosis.

## 1. Introduction

Adenomyosis is characterized by the extension of endometrium into the myometrium, along with myometrial smooth muscle cells hyperplasia and hypertrophy. It is common in women of reproductive age and often regresses after menopause [1, 2]. Estrogen plays a crucial role in the pathogenesis of adenomyosis. The effects of estrogen are mediated by two types of estrogen receptors: nuclear estrogen receptors (ER-*α* and ER-*β*), which are members of the nuclear receptor family of intracellular receptors, and membrane estrogen receptor (GPER or G-protein-coupled estrogen receptor) [3]. Adenomyosis is known to be associated with changes in the expression of ER-*α* and ER-*β*. A recent study showed that ER-*α* expression was reduced in the mid-secretory phase endometrium of women with adenomyosis, whereas ER-*β* was increased not only in the endometrium but also in the inner myometrium and outer myometrium of women with adenomyosis compared with control subjects [4]. However, the expression of GPER in the uterus of women with adenomyosis has not been previously investigated. In this study, we studied the expression of GPER in the outer and inner myometrium (junctional zone) of women with adenomyosis and compared the results to a group of control subjects.

## 2. Materials and Methods

### 2.1. Subjects

All subjects recruited in this study were premenopausal women who underwent hysterectomy at the Beijing Obstetrics and Gynecology Hospital, Capital Medical University, Beijing, China.

The inclusion criteria included the following:

1. Regular 25–35 days' cycles
2. No hormonal treatment or having not used an intrauterine device for 3 months prior to hysterectomy
3. No evidence of leiomyoma.

There were 2 groups of women recruited. Group I consisted of 42 women with adenomyosis, histologically confirmed. In this study, the diagnosis of adenomyosis was based on histological confirmation of the presence of endometrial tissue more than 2.5 mm below the endomyometrial junction or a JZ thickness of more than 12 mm [5]. The additional inclusion criterion for women in this group was the presence of dysmenorrhea with or without menorrhagia prior to the surgery. In this respect, we have included only subjects with symptomatic adenomyosis, which was the underlying reason for their request for hysterectomy. Group II consisted of 34 subjects with no evidence of adenomyosis who underwent hysterectomy due to early cervical cancer or ovarian cancer. The exclusion criteria for this group of women were preoperative radiotherapy or chemotherapy and involvement of the endometrium or myometrium by neoplasm. As the study was based on histological confirmation of adenomyosis on hysterectomy specimens and the collection of biopsy from different parts of the excised uterine specimen, it was necessary to recruit control subjects who required hysterectomy for a different clinical reason. However, it was also considered necessary to exclude uterus with significant myometrial pathology such as myoma which could have altered histological findings in the junctional zone or myometrium. Given that it was unusual for women of reproductive age to have a normal uterus removed other than those who suffered from early cervical or ovarian cancer, we have chosen to include this group of women as control subjects. As it happened, all subjects included in the study belonged to early cervical cancer.

Excisional biopsy of the junctional zone and outer 1/3 of the myometrium was performed from the anterior fundal wall of each hysterectomy specimen. The biopsy of the outer 1/3 myometrium was undertaken from 2 mm beneath the serosal surface of the uterus, whereas the biopsy from the junctional zone was undertaken 2-3 mm beneath the endometrium, taking special care not to include any normal endometrial tissue in the biopsy.

### 2.2. Histological Dating

Histological dating of the endometrium from the uterine specimens was performed by an experienced pathologist with the use of standard criteria [6]. The specimens were then classified into proliferative (PP) or secretory phases (SP).

### 2.3. Western Blotting

Homogenized tissues were lysed for protein extractions. Protein extracts (30–50 mg) were separated by electrophoresis on 12% sodium dodecyl sulfate-polyacrylamide gels. Proteins were transferred to polyvinylidene fluoride (PVDF) membranes; incubated with rabbit anti-GPER (ab39742; from Abcam, Cambridge, Massachusetts) as a primary antibody; and then developed with a secondary anti-rabbit antibody (obtained from Cell Signaling Technology, Danvers, Massachusetts). The signal was detected using a LI-COR Biosciences Odyssey Infrared Imaging System (LI-COR, Lincoln, Nebraska). Equivalent protein loading and transfer efficiency were verified by staining for GAPDH (D16H11, from Cell Signaling Technology, Danvers, Massachusetts).

### 2.4. Quantitative Real-Time Polymerase Chain Reaction

Total RNA was extracted and purified from the tissue specimens. 1.0 *μ*g RNA was reverse transcribed with random primers using Superscript II reverse transcriptase (Life Technologies, Inc., Melbourne, Australia). Quantitative PCR was performed in the presence of SYBR Green (Tiangen Biotech, China), and amplicon yield was monitored during cycling in an ABI 7500 Real-Time Polymerase Chain Reaction System (Applied Biosystems, Grand Island, New York) that continually measured fluorescence caused by the binding of the dye to double-stranded DNA. The cycling conditions were 95°C for 15 minutes, 40 cycles at 95°C for 10 seconds, and 60°C for 32 seconds. The cycle at which the fluorescence reached a set threshold (cycle threshold) was used for quantitative analyses. The cycle threshold in each assay was set at a level at which the exponential increase in amplicon abundance was approximately parallel between all samples. Relative gene expression was calculated in relation to an internal control for normalization (glyceraldehyde 3-phosphate dehydrogenase, GAPDH), using the comparative cycle threshold method. Primer sequences for GPER and GAPDH are presented in Table 1.

### 2.5. Ethics

This study was approved by Ethical Committee of Clinical Research of Beijing Obstetrics and Gynecology Hospital, Capital Medical University, Beijing, China. Written informed consent was obtained from all patients who participated in this study. This study was conducted in accordance with the Declaration of Helsinki (1964).

### 2.6. Statistical Analysis

Normally distributed data were presented as mean ± standard deviation. Student* t*-test was used to test for differences between groups. Statistical analyses were performed with the use of SPSS 19.0 for Windows. For all tests, a *p* value of <0.05 was considered statistically significant.

## 3. Results

### 3.1. Demographics

A total of 42 women with adenomyosis and 34 women without adenomyosis (control subjects) were included in the study. In group I, 23 specimens were classified as proliferative and 19 as secretory. In group II, 16 specimens were classified as proliferative and the remaining 18 as secretory. The demographic details of these two groups of women are compared in Table 2. There was no significant difference in any of the features between the two groups.

**Figure 1:**
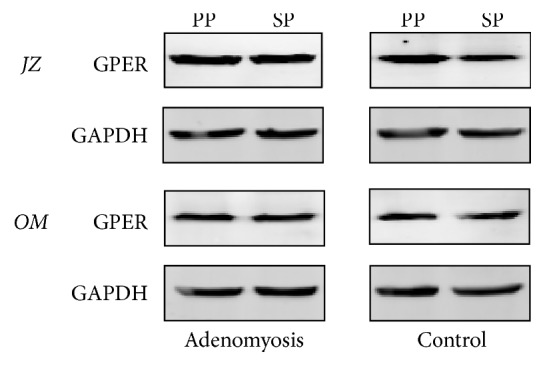
Representative western blotting of G-protein-coupled estrogen receptor (GPER) and glyceraldehyde-3-phosphate dehydrogenase (GAPDH) in biopsy specimens from the junctional zone (JZ) and outer myometrium (OM) in the proliferative phase (PP) and secretory phase (SP).

Using western blotting, GPER protein expression signals appeared at approximately 42 kd (Figure 1). The position of the GPER signal was the same as that observed in MCF-7 breast cancer cells (data not shown).

### 3.2. Comparison of GPER Expression between Proliferative Phase and Secretory Phase

In Table 3, the GPER protein and mRNA expression in the proliferative and secretory phase were compared, separately in women with and without adenomyosis, and in the JZ and OM.

In the control subjects, the expressions of both protein and mRNA in the JZ in the proliferative phase were both significantly (*p* < 0.05) higher than those of the secretory phase, whereas the expressions of both protein and mRNA in the OM in the proliferative phase and the secretory phase were similar.

In women with adenomyosis, in contrast, there was no significant difference in the expression of GPER protein and mRNA level between the proliferative phase and secretory phase in both the JZ and OM.

### 3.3. Comparison of GPER Expression between Junctional Zone and Outer Myometrium

In Table 3, the GPER protein and mRNA expression in the junctional zone and outer myometrium were compared, separately in women with and without adenomyosis and in the proliferative and secretory phases.

In the control group, in the proliferative phase, the expression of both protein and mRNA in the JZ was higher than that of the OM. In the secretory phase, however, the expression of both protein and mRNA in the JZ was similar to that of the OM.

In women with adenomyosis, on the other hand, the expression of both protein and mRNA in the JZ were higher than that of the OM in both the proliferative and secretory phases.

### 3.4. Comparison of GPER Expression between Adenomyosis and Control Groups

In Table 3, the GPER protein and mRNA expression in women with and without adenomyosis were compared, separately in the junctional zone and outer myometrium and in the proliferative and secretory phase.

In the JZ, the GPER protein expression and the mRNA level in women with adenomyosis were significantly higher than that of control subjects in both the proliferative phase and secretory phase.

In the OM, the GPER protein expression and the mRNA level in women with adenomyosis were also significantly higher than that of control subjects in both the proliferative phase and secretory phase.

## 4. Discussion

In this study, we found that the expression of GPER protein and mRNA in women with adenomyosis was significantly higher than that of women without adenomyosis, both in the junctional zone and in the outer myometrium and both in the proliferative and in the secretory phases.

Adenomyosis is often considered to be an estrogen-dependent disease with changes in structure and function of the JZ [1] observed in the majority of cases. The JZ is responsible for uterine peristalsis which regulates sperm transport and implantation [7, 8]. Structural changes in the JZ in adenomyosis commonly include JZ hyperplasia and thickening [9]. Ultrastructurally, the myocytes in the JZ of uterus affected by adenomyosis appeared smaller [10]. Abnormal expression of oxytocin receptor in the JZ of women with adenomyosis has been observed [11], which may explain the occurrence of hyperperistalsis and dysperistalsis, in turn leading to dysmenorrhea and disturbance of reproductive function. However, the outer myometrium, whose primary function is involved with parturition [12], is often not affected in adenomyosis until a later stage.

There are two different types of estrogen receptors. The nuclear estrogen receptors (ER-*α* and ER-*β*) mediate gene expression through binding to estrogen receptor elements in the promotor and regulatory regions of the target genes; in contrast, GPER, the other type of estrogen receptor, mediates rapid cellular effects. GPER is a membrane estrogen receptor, a 7-transmembrane spanning G-protein-coupled receptor, also called G-protein-coupled receptor 30 (GPR30) [13]. GPER is structurally and genetically unrelated to ER-*α* and ER-*β* and expressed independently [3]. It binds to estrogen with high affinity, whereas binding affinities of GPER for other steroid hormones are very low [14]. In earlier studies, estrogen effects can be mimicked by selective GPER agonist or antagonist, whereas, in GPER knockout mice, these effects are absent or reduced, suggesting that GPER plays an essential role [15]. Expression of GPER has been described in multiple physiological systems and tissues, including the breast, heart, endothelium, brain, bone, adrenal, kidney, endometrium, and ovary [15–20]. The changes in GPER expression in a number of gynecological conditions such as the endometrium of women with endometriosis [21] and smooth muscle of myoma [22] have been examined. On the other hand, whilst the expressions of nuclear estrogen receptors (ER-*α* and ER-*β*) have been examined in the inner and outer myometrium of adenomyosis [12], the expression of GPER in adenomyosis has not previously been studied. Evidence has shown that GPER agonist G1 can lead to apoptosis in endometriosis and suppress proliferation of endometriotic stromal cells [23]. GPER is closely related to the outcome of estrogen therapy on various estrogen-dependent diseases [24–26] and selective GPER ligands (such as GPER agonist G1 and antagonist G15) have been shown to exert control of these diseases [27–29]. A better understanding of the role played by GPER in the pathogenesis of adenomyosis may open up opportunity for GPER-targeted therapy for the condition [15].

The special design of this study enabled us to examine the cyclical change and anatomical variation of GPER expression in control subjects, in addition to comparing GPER expression between women with and without adenomyosis. One possible limitation of our study was that biopsy specimens from the junctional zone of women with adenomyosis often contained foci of endometrial tissue which could have contributed to the observed difference in results between the two groups of subjects.

*Cyclical Changes. *In our study, in control subjects, we observed that the expression of GPER in the proliferative phase in the JZ was higher than that of secretory phase of the JZ; however the cyclical change was not observed in the OM. In women with adenomyosis, the cyclical change appeared to have disappeared. Similar cyclical changes in the ultrastructure of myocytes in the JZ and OM were observed in our previous study [10].

*Anatomical Variation. *As for the anatomical variation, in control subjects, the expression of GPER in the JZ was higher than that of the OM in the proliferative phase but not the secretory phases. In contrast, in women with adenomyosis, the expression of GPER in the JZ was higher than that of the OM in both the proliferative phase and the secretory phases. In other words, the anatomical variation between the JZ and OM in women with adenomyosis was consistently observed, independent of the phases of the cycle.

*Adenomyosis versus Control. *In contrast to the observations relating to the cyclical and anatomical variation of GPER in which significant difference in control subjects was found only in the proliferative phase and JZ, there was consistent and significant difference in GPER expression regardless of the stage of the cycle (proliferative or secretory) or anatomy (JZ or OM).

Taken together, the cyclical and anatomical variation of GPER observed in control subjects in this study is consistent with our current understanding that the effects of estrogen are more dominant in the proliferative phase than the secretory phase, and more pronounced in the JZ than in the OM. In addition, the special design of our study enabled us to control for two important confounding variables (cyclical changes and anatomical variation); by doing so, the variance in results due to the effect of these confounding variables was reduced. Whilst it is already known that the expressions of estrogen nuclear receptors ER-*β* (but not ER-*α*) and progesterone receptor are different between women with and without adenomyosis [4], the additional finding in this study that GPER expression is also altered in women with adenomyosis confirms the notion that adenomyosis is associated with alteration in several different steroid receptors.

Overall, the finding observed in this study appears to have provided a molecular basis for the smooth muscle hyperplasia and hypertrophy observed in adenomyosis [1, 2]. Specifically, it seems plausible that the abnormally elevated GPER expression in the JZ is one mechanism by which the smooth muscle cells continue to proliferate in adenomyosis.

To conclude, we have found significant and consistent increase in GPER expression in adenomyosis, in both proliferative and secretory phases and in both the JZ and OM, suggesting that GPER plays an important role in the pathogenesis of the condition. It remains to be seen if treatment targeting the expression of GPER by the use of selective GPER ligands may help to treat or prevent the condition.

## Figures and Tables

**Table 1:** Primer sequences for GPER.

	Primer	Sequence
GPER	Forward	5′-TGACCATCCCCGACCTGTAC-3′
	Reverse	5′-CAGGTGAGGAAGAAGACGCT-3′
GAPDH	Forward	5′-CTCCTCCACCTTTGACGCTG- 3′
	Reverse	5′-TCCTCTTGTGCTCTTGCTGG-3′

**Table 2:** Demographic details of patients with adenomyosis and control subjects.

	Adenomyosis	Control	*p* value
Age (years)	44.1 ± 2.9	43.0 ± 4.4	0.07
Parity	1.3 ± 0.1	1.4 ± 0.1	0.45
Miscarriage	2.1 ± 1.4	1.8 ± 1.1	0.06
Body mass index, kg/m^2^	23.2 ± 2.7	24.1 ± 3.3	0.09

Values are given in mean ± standard deviation.

**Table 3:** Relative GPER protein and mRNA expression levels (GPER/GAPDH).

	Adenomyosis group (*n* = 42)		Control group (*n* = 34)	
	JZ	OM	JZ	OM
Relative GPER protein expression levels				
PP	1.12 ± 0.072^b,c,e^ (*n* = 23)	0.96 ± 0.043^b,c,e^ (*n* = 23)	0.95 ± 0.027^a,c,e^ (*n* = 16)	0.83 ± 0.051^b,c,e^ (*n* = 16)
SP	1.09 ± 0.076^b,c,e^ (*n* = 19)	0.94 ± 0.052^b,c,e^ (*n* = 19)	0.82 ± 0.097^a,d,e^ (*n* = 18)	0.84 ± 0.086^b,d,e^ (*n* = 18)
Relative GPER mRNA expression levels				
PP	1.52 ± 0.12^b,c,e^ (*n* = 23)	1.31 ± 0.09^b,c,e^ (*n* = 23)	1.28 ± 0.07^a,c,e^ (*n* = 16)	1.04 ± 0.1^b,c,e^ (*n* = 16)
SP	1.51 ± 0.14^b,c,e^ (*n* = 19)	1.29 ± 0.18^b,c,e^ (*n* = 19)	1.09 ± 0.13^a,d,e^ (*n* = 18)	1^b,d,e^ (*n* = 18)

Values expressed as mean ± standard deviation. ^a^Comparison between proliferative phase and secretary phase (same group and zone), *p* < 0.05, Student *t*-test; ^b^comparison between proliferative phase and secretary phase (same group and zone), *p* ≥ 0.05, Student *t*-test; ^c^comparison between outer myometrial zone and junctional zone (same group and phase) *p* < 0.05, Student *t*-test; ^d^comparison between outer myometrial zone and junctional zone (same group and phase) *p* ≥ 0.05, Student *t*-test; ^e^comparison between adenomyosis and control group (corresponding phase and zone) *p* < 0.05, Student *t*-test.
